# The Moral Aspects of Medical Life; Consisting of the "Akesios" of Professor H. F. K. Marx

**Published:** 1847-01-01

**Authors:** 


I. The Moral Aspects of Medical Life; consisting of the "Akesios" of Professor K. F. H. Marx. Translated from the German, with Biographical Notices and Illustrative Remarks, by *James Mackness*, M.D. Consulting Physician to the Hastings Dispensary, &c. &c. Small 8vo. pp. 348. London: Churchill, 1846.

II. Life of George Cheyne, M.D. With Extracts from his Works and Correspondence. 12mo, pp. 141. Oxford, 1846.

The first of these works is a very interesting and instructive volume, intended and well calculated to do good to the profession. We sincerely trust that the notice, which we are about to give of its contents, may serve to make it generally known, and may thus induce most of our readers to possess themselves of the book itself. The German original, entitled, *Akesios; Blicke in die ethischen Beziehungen der Medicin, von K. F. H. Marx,* was published about two years ago at Gottingen. It consists of a series of letters addressed to various deceased physicians, the prominent features and events of whose lives, being the subjects of each discourse, are made to afford a pleasing vehicle for a variety of professional and moral reflections. The literary reader will therefore see that the plan is somewhat similar to that which Landor has adopted, with so much success, in his well-known "Imaginary Conversations."

The first intention of Dr. Mackness was simply to translate the Akesios; but, struck with the fine observations and the many germs of noble thought which often lay buried in a single sentence, he was happily led to extend the plan of his work, by prefixing a biographical notice to each letter, and appending illustrative observations in the way of commentary on its leading thoughts—until it at length acquired its present form.

The meaning of the title which Professor Marx has given his production, and the design which he had in view, are thus explained in his own preface:—

"Ακεσιος, or the Healer (*ακεομαι medeor*) was one of the names by which the Greeks designated the demi-god whom the Egyptians called Harpocrates. His birth, which was placed in the winter solstice, indicated the feebleness of the wintry sun, whilst it left hopes, also, of his return in spring, to spread new life. Thus was the god at once an emblem of the infirmities of the sick, and of their hopes of recovery. He was also depicted holding his finger to his lips, symbolizing that sacred silence concerning the mysteries of medicine ever required of the initiated.

"I restricted myself, in the following pages, to the former cheering and consolitary symbol. This work is designed to discuss weighty points in the healing art as it now exists. As to mysteries,—if, in fact, there be properly any such in medicine,—they will not here be unravelled. I deal not in this work with systems of healing, or methods of treatment;—no, what dwells in every heart, and is visible to every eye, and yet is intimately connected with the medical profession, the ordinary, the ethical, the individually personal, this, this alone is my subject. That I have thrown it into the form of letters was for this reason, that I wished, through the medium of certain individuals distinguished, at least, if not universally known, as masters in their particular department, to give prominence to those peculiar characteristics which were exhibited in their practice, their lot in life, or their self-confessions. These persons are no longer living, most of them, indeed, belong to times long since gone by—a circumstance which afforded me all the freer scope for selection, and if the reader does not object to the design of these letters, he will, no doubt, readily excuse the fiction by which I have allowed myself to propound questions and difficulties mooted amongst the living, and to decide upon them through the medium of the departed." P. 2.

The letters are twelve in number, and are addressed to the following characters—Stieglitz, Petrus de Apono, Dr. Cheyne, Hallé, Dr. James Gregory, Albert Thaer, Dr. Lettsom, Nicholas Tulpius, Pinel, Dr. Mead, Desgenettes, and Boerhaave. We shall select for our notice three or four of the most distinguished names on this list, beginning with the Hippocrates of the Dutch School, the great and good Professor of Leyden.

HERMAN BOERHAAVE was born at Voorhout, near Leyden, in the year 1668. In early life, he made great proficiency in classical literature; but his progress was checked, in his 12th year, by one of those *apparent* (truly it may be called so) calamities, which, by their sobering and deepening work on the mind, often prepare the character for future eminence. This calamity was the breaking out of a malignant ulcer upon his left thigh, which, for five years, defied the power of the healing art, as it was then known. From this affliction he gained two things—a feeling sense of the sufferings of the sick, and an experimental conviction of the insufficiency of existing modes of practice. After all other means had failed, he cured himself by fomenting the part with salt and urine.

In his 14th year, Boerhaave lost his father, who, a minister himself, had designed to bring up his son to the clerical profession. Soon after this period, he was sent to the University of Leyden, where he continued for several years, and distinguished himself by his talents and assiduity. Mead was his fellow-pupil, and the celebrated Dr. Pitcairn was their preceptor.

In 1690, he took his degree in philosophy. His pecuniary means being then very low, he began to lecture on mathematics, on which he had bestowed great attention, to a select number of pupils, and was thereby enabled to defray his current expenses, and to continue the prosecution of his studies at the same time. Among these, Medicine was one for which he had the highest admiration; and, although still contemplating the ministry as his future calling in life, he determined to make himself thoroughly acquainted with the art of physic, before he took upon himself any clerical duties. On this determination, Dr. Johnson very justly remarks, in his Life of Boerhaave, "that Providence seldom sends any into the world with an inclination to attempt great things who have not abilities likewise to perform them. To have formed the design of gaining a complete knowledge of medicine, by way of a digression from theological studies, would have been little less than madness in most men, and would have only exposed them to ridicule and contempt. But Boerhaave was one of those mighty geniuses to whom scarce anything appears impossible, and who think nothing worthy of their efforts but what appears insurmountable to common understandings."

It is interesting to know that Hippocrates and Sydenham were the two authors, whose writings chiefly attracted the attention of Boerhaave. In 1693, he took the degree of Doctor of physic. The subject of his thesis was *De utilitate explorandorum excrementorum in ægris, ut signorum.*

It was now his intention to devote himself entirely to the labours of the ministry.

> "But Providence had designed otherwise, and made use of a false and malicious report to turn the current of his activity into that channel in which it could be most useful. Whilst sitting in a common passage-boat, or *treikschuyt*, between Leyden and some adjacent place, several of the passengers were discussing the views of Spinosa, then newly brought before the public, and one person in particular was loud and bitter in his condemnation of them. Boerhave, though he had on other occasions refuted these views, and though his own opinions were deeply Christian, was yet led by impartial love of justice, calmly to inquire of the speaker whether he had ever read the works of Spinosa. The stranger answered he had not, and should esteem it wicked even to look into them. 'How, then,' said Boerhaave, 'can you pretend to judge of them?' This reproof silenced the declaimer, but a report was circulated by some present that Boerhaave was himself a follower of Spinosa, and from this bigoted and utterly groundless charge obstacles were raised, which barred his entrance into the sacred profession, and threw him of necessity into one only less sacred than that which he had coveted." P. 317.

His practice being but inconsiderable for several years, he had the more time—and well did he employ his leisure—for carrying on his favourite pursuits; studying, making chemical experiments, and, in short, exploring every mine of medical lore. Nor did he forget the higher duty of deep and thoughtful perusal of the Word of Truth. "Early on every morning throughout his busy life, he retired for an hour for prayer and meditation on the Scriptures. Here, according to his own avowal, he sought and found strength for the duties of the day, and thus was enabled with vigour and cheerfulness to go through his great amount of daily business. In his hours of relaxation his conversation frequently turned on the excellence of the Christian religion, and the value and authority of the Scriptures, delighting to recommend to others what he so highly prized. Health, he was accustomed to say, must be promoted by the tranquillity of the mind, and he knew of nothing which could support himself or his fellow-creatures in the calamities of life but a well-grounded confidence of God."

In 1701, he was elected to the chair of the Institutes of Physic in the university of Leyden. Being an eloquent speaker, and deeply versed in all the knowledge of the time, his reputation soon became generally known. In 1714, he had attained the highest honours in the university, and was then also appointed physician to St. Augustine's hospital in Leyden.

In consequence of his early predilection for mathematical, and subsequently for chemical, pursuits, Boerhaave, notwithstanding his ardent admiration of the works of Hippocrates and Sydenham—the two very men whose writings were less imbued with theory than those of any other medical authors—could not emancipate himself from the then prevailing doctrines in reference to disease. Not satisfied, however, with any of the systems that had been proposed, he endeavoured to combine in one and the same theory the vital physiology of Hippocrates, the chemical principles of Silvius, the mechanical views of Borelli and Bellini, besides many other fanciful incongruities ; attributing, however, decidedly more to the chemical and mechanical forces, than to the more secret and mysterious powers of life.

"Thus the calibre of the vessels adjusted to the dimensions of the globules composing the liquids of the body formed, according to him, the hydraulic relation, on which depended the circulation of the humours, their separation from the blood in the different secretory organs, the morbific congestion of the blood in various fluxions, in humours, inflammations, and the like, and hence he concluded that all the efforts of the physician should be directed to establish this relation, or rather mechanical equilibrium. Nor did he stop even here. To the mechanical hypotheses just mentioned he added others, founded on chemical principles, when, in attempting to explain the causes and the phenomena of diseases, he admitted the formation of pretended acrimanies in the blood, which the physician ought, according to him, to have constantly in view in order to neutralize them—acrimonies which were long famous in the language of the schools, and which are still found in that of ordinary life. The whole phenomena of diseases, with the spontaneous evacuations by which they are terminated, and which constitute the crisis, find a ready explanation on this vicious system, which seems to offer a reason, when it only mystifies with a word involving a gratuitous hypothesis. In practice, however, theory receives many modifications, and there can be little donbt that in prescribing for patients, Boerhaave was more guided by experience and good sense than by the strongly eclectic doctrine to which we have alluded."* P. 320.

This system continued to be dominant in the schools for many years after the death of its propounder, and so high was the reputation of the professor that the university of Leyden was long regarded as *the* school in Europe for the sciences of medicine, chemistry, and botany. It is recorded that the Czar Peter did not consider his education complete, until he had attended the lectures of Boerhaave. His fame reached even to the Celestial Empire; for we are told that a letter was once addressed to him from a mandarin, having the superscription, "To the illustrious Boerhaave, physician in Europe :"—it did not fail to reach its destination.

In 1722, he was confined to bed for five months with a severe fit of the gout. Five years afterwards, he had a violent attack of fever ; and the complaint returning at intervals, he felt himself obliged to resign the professorships of Chemistry and Botany. His time, however, continued to be fully occupied with his professional labours, letters being addressed to him from all parts of Europe by those who were unable to consult him personally. In spite of his erroneous theory of diseases, his practice appears to have been highly esteemed, and all acknowledge that his sagacity and penetration in diagnosis were very remarkable.

It was in the year 1737 that he felt the first approaches of that disorder, which was to prove fatal. The symptoms, which he himself describes in a letter addressed to a friend in London, too well attest the nature of the malady. The following extract cannot but be read with interest, not only

---

* Encyclopædia Britannica.

as a faithful description of disease, but as a beautiful specimen of Christian fortitude and resignation.

"Ætas, labor, corporisque opima pinguetudo, effecerant, ante annum, ut inertibus refertum, grave, hebes, plenitudine turgens corpus, anhelum ad motos minimos, cum sensu suffocationis, pulsu mirificè anomalo, ineptum evaderet, ad ullum motum. Urgebat præcipuè subsistens prorsus et intercepta respiratio ad prima somni initia; unde somnus prorsus prohibebatur, cum formidabili strangulationis molestiâ. Hinc hydrops pedum, crurum, femorum, scroti, præputii, et abdominis; quæ tamen omnia sublata. Sed dolor manet in abdomine, cum anxietate summâ, anhelitu suffocante, et debilitate incredibili; somno pauco, coque vago, per somnia turbatissimo; animus verò rebus agendis impar. *Cum his luctor fessus, nec emergo; patienter expectans. Dei jussa, quibus resigno data, quæ sola amo, et honoro unicè.*" P. 323.

Need we say that he died the death of the righteous? He expired on the 23rd September 1738, in the 70th year of his age. The city of Leyden raised a monument to his memory, in the church of St. Peter, with this inscription: *Salutifero Boerhaavi genio sacrum.* It bears a medallion of him, and affixed to this is a ribbon which displays his favourite motto, *Simplex sigillum veri.*

The principal works of Boerhaave are his "Institutiones Medicæ," "Aphorismi de cognoscendis et Curandis Morbis," on which Van Swieten published Commentaries in 5 vols. 4to., and "Libellus de Materia Medica et Remediorum Formulis quæ serviunt Aphorismos."

Boerhaave was a man of very lofty intellectual and moral attainments. No one ever more truly exemplified those graces of character so beautifully described by the Roman satirist:

> Compositum jus, fasque animi; sanctosque recessus
> Mentis, et incoctum generoso pectus honesto.

Conscious of his own rectitude, he always refused to take any notice of slander and abuse. "They are sparks," he used to say, "which will go out of themselves, if you do not blow them." And again: "The surest remedy against scandal is to live it down by perseverance in well-doing, and by praying to God that He would cure the distempered minds of those who traduce and injure us." Pure and noble sentiments! Well would it be if all medical men realized the truth by their own conduct. If they would seek to do so, let them not forget that they must go for instruction where Boerhaave sought and found it; it is expressly said by his biographer that "his deep sense of religion and his devotional habits constituted the basis of his character and the springs of all his virtues." Is it necessary to add that he had solemn thoughts of medical responsibility? He used to say that, where there had been any inattention or negligence, the life of the patient would be required at the physician's hands.*

---

* The venerable Hufeland has given utterance to a similar thought. "Remember," writes the late Nestor of German medicine, addressing each individual of the medical profession, "what thou art, and what thou shalt be. Thou hast been appointed by God a priest of the holy flame of life, a curator and dispenser of His highest gifts, health and life, and of the hidden powers which He has laid up in nature for the welfare of man. A high and holy vocation! Exercise it aright, not for thy own profit nor yet for thy own praise, but for the glory of God, and for the benefit of thy neighbour. Hereafter, thou must render an account of thy mission."—*Enchir. Med. f.* 80.

With this biographical sketch before him, the reader will be now prepared to appreciate the force and beauty of the following extracts from Professor Marx's "Letter to Herman Boerhaave."

"'Simplicity is the seal of Truth,' is in the mouths of thousands who do not belong to the profession. This beautiful and characteristic maxim is the proximate cause of this Letter.

"I am of opinion that earthly action can aim at no higher object than simplicity in thought and deed, and that existence beyond this present world must bring this aim to fuller perfection.

"The physician, like the surgeon, cannot be too simple. If medical science is not to be considered merely as a part of natural history, in which men are to be studied like plants, their properties and strength to be ascertained, by means of microscopes, chemical reagents, and anatomical knives, for the satisfaction of the curious, but as destined under all circumstances to alleviate suffering, lighten and support life, the grand problem consists in this—to search out humanity in all its aspects, and to adapt all remedies as simply as possible to the doing of good.

"Since you understood so well how to blend clearness, logical order, and acute perception with simple truth in all your actions and opinions, I would willingly speak to you on several points, which, in spite of the extension of our knowledge, have not yet attained that plain, regular character in which you would have fixed them.

"In order to attain perfect correctness, it is, as you may easily conjecture, first of all necessary that we should understand one another's language, and that this should remain unalterable as regards the conventional symbols for definite objects and ideas; in our own times, however, we quarrel much about words, and many a one who has to remember in a definition an unimportant improvement, strikes out also a new, singularly-sounding word, thinking he has by that means essentially advanced the matter.

"In the department in which you were once the head, you would now scarcely know yourself; the most familiar things would seem to you strange, and even the student would laugh at your ignorance.

"For fainting, you would hear of *anencéphalohemia;* for apoplexy, *homœncephalorrhagia;* for diabetes, *hyperurorrhœa;* for catarrh, *rhinite;** for mental derangement, *cerebria;*† for inflammation, *hematelangiosa.*"‡ P. 325-6.

"But you must not judge of the labours of our time from these and similar examples. As Morgagni‖ loved the simple, so is it still the case with the ablest men, even in respect of the names of objects. It is the novice, not the master, who attaches value to such trifles. The master concerns himself for the kernel, not for the shell, the permanent, not the transitory. The single flower may blow unobserved beside the double, but it is the single alone which produces the seed, the other perishes in its beauty." P. 327.

Professor Marx then shows by a multitude of instances that, in all departments of the art of Physic, as well as in Pharmacy, Chemistry and other branches of science, the tendency has been, during the present century, to simplify what is complicate, and to educe general principles from a multitude of details.

---

* "See for the like, the Nomenclature Organo Pathologique of Piorry. Traité de Diagnostic et de Semeiologie, Paris, 1837, 8vo."

† "Scipion Pinel, Physiologie de l'Homme aliéné, Paris, 1833, 8vo."

‡ "J. F. Lobstein, Essai d'une nouvelle Théorie des Maladies, Strasburg, 1835, 8vo."

‖ "A. Fabronii, Vitae Italorum, Romæ, 1769, 8vo. p. 328.

" Simplifying is spiritualizing; it is to raise objects to right thought and comprehension.

" The more the materials of observation, as of remedies, increase, the more necessary it is to introduce order and generalization into the whole, which can only be attained by simplicity.

" Thus you proceded in your efforts, directed to the just and enduring, simply to reduce medicine to the smallest possible quantities, but of well-established ingredients.*

" As long as the mind is wearied with the manifold and the dissimilar, it will be easily overpowered by the mass. If, however, it succeeds in mastering the leading and governing idea, it soon finds the shortest and nearest way out of the entangled maze.

" A physician whose endeavours are thus directed, will, for the most part, be enabled to maintain composure and clearness of thought in sudden attacks of disease.

" From the complication of pressing symptoms he directs the mental eye to the ascertained, the safe, and certain principles, of diagnosis and treatment: like the Zeus of Homer, who, amidst the clamor of the Trojan combatants, turned his eyes on the peaceful labours of an inoffensive peasantry.

" The acquirement of such a habit of thinking and acting has an important influence on the whole plan of life." P. 331.

And then, passing from the science of Matter to that of Mind, we come to these beautiful remarks :—

" Even in morals and manners, the simplest natures are ever the noblest, the strongest. They need no artificial excitement to enliven existence, or to develop their own hidden qualities.

" As in the great circle of existence all happens from small uncomplicated forms and laws, so the higher spiritual existence acknowledges in its impressions and realizations a simple characteristic, whilst it bears on itself the stamp of truth, suggesting and refreshing all minds which come in contact with it. Thus in music, the simple chords please the ear the most; thus the pure light, which penetrates the clear fountain or the transparent crystal, is more refreshing than that which streams through the many-coloured prism.

" The wise man lives, speaks, and acts with simplicity; the fool cannot employ variety enough. Quickness in decision and certainty in action, are allied to definitiveness and clearness of expression. For accuracy of perception, and the ability to be at all times free and ready for action, originate thence in an immeasurable degree.

" Our idea of the soul must be as the perfection of simplicity: the highest with which man in his thoughts and deeds, his wishes and knowledge, has to strive, and which also first imparts the right sanction to the richest internal worth is simplicity. It is the measure as the test of right, and, as you admirably say, the Seal of Truth."† P. 333.

The opening remarks of Dr. Mackness on this letter to Boerhaave are

---

* " H. Boerhaav: Praelect. Acad. ed. Alb. Haller i, p. 43: 'Medicus Italus misit ad me exiguum libellum, cui titulum fecerat, 'Picola Arte Medica.' In eo congesta erant in breve compendium, quaecunque indubitatæ fidei propositiones apud Medicos possunt pro axiomatibus haberi. Ad idem studium excito vos, quantum possum, auditores; non alia ratione longius proferetis artem."

† " Even in antiquity the saying was valued :—

Απλοῦς ὁ μῦθος τῆς ἀληθειας ἐθυ.

*Euripid.* Phœniss. 472."

characterised not less by the appropriateness of the sentiments than by the gracefulness of the language.

"Simplicity is stamped upon all the works of God in creation. The normal type of animal life is a simple ovum; the commencement of vegetable life is a simple cell; and from these ova and cells all the varied and complicated forms of vegetable and animal matter emanate. The simple law of attraction holds the earth in its orbit, and determines the motions of the planets: the same law, variously modified, influences the form of every part of the material universe. If we get into the world of mind and spirit, simplicity still meets us; and thus in religion the Gospel—the Divine plan of salvation—is simple, as Cowper has beautifully sung:

"Oh! how unlike the complex works of man,
Heaven's easy, artless, unencumber'd plan:
No meretricious graces to beguile,
No clustering ornaments to clog the pile.
From ostentation as from weakness free,
It stands, like the cerulean arch we see,
Majestic in its own simplicity."

"The expression of true feeling is simple; children and persons under strong excitement, usually speak in simple language. The simplest poetry is ever the most really sublime. Marx has an exquisite illustration of the beauty of simplicity in his instance of the single, not the double flower, being that which bears the fruit; and as it is with reference to medical studies, to medical practice, and to medical life that he writes, let us inquire how far in these matters simplicity is indeed the seal of truth." P. 334.

"There can be no doubt that the single element of simplicity of purpose, the simple aim to do our duty, the simple reliance upon Divine support and guidance, would strike at the root of all the faults and follies observable in manners and deportment, and would go further towards forming an easy and agreeable address than all the maxims of Chesterfield. For what is it that most frequently renders the manners of persons disagreeable? It is something which arises from the hidden root of *self-seeking;* either affectation, pedantry, egotism, self-sufficiency, assumption, or superciliousness." P. 341.

That simple but most comprehensive maxim of Christian morality, "Do unto others as you would that they should do unto you," is the foundation of all sound professional ethics. No physician can be much at a loss how to conduct himself, either to patients or to his professional brethren, if he follows this as the guide of his life. Well has Professor Marx observed, that "Medical Science is not merely a part of Natural History; it is not a mere field for curious investigation, an arena for the sharpening of men's wits by ingenious theories and elaborate discussions; it has a momentous responsibility, an important practical aim, that aim being, under all circumstances, to alleviate suffering, lighten and support life. Its grand problem therefore is, to search out humanity in all its aspects, and to adapt all remedies as simply as possible to the doing of good."

Would that the truth of these remarks were more deeply felt and more openly recognised by the profession at large!—in private as well as in public, in our dispensaries and hospitals, in our books and journals, in our meetings and associations, in our schools and colleges, in the every-day walk of every individual; each and all of us remembering that we are but as missionaries and messengers in the hands of Him, one of whose great offices it is "to heal the sick, to open the eyes of the blind, and to speak comfort to those that are afflicted."*

Recurring to Boerhaave's favourite motto, that "simplicity is the seal of truth," Dr. Mackness closes his comments on the letter to him in these words:

> "May we then learn to be simple; simple in our *object,* simple in our *views,* simple in our *practice,* simple in our *habits,* simple in word and deed. But whilst we aim at simplicity, let us take care that we have truth for our foundation; for many systems and theories are apparently very simple, but being based on insufficient evidence, they are wanting in the important element of truth. There is no doubt but that truth is in itself always simple, but in our limited knowledge we may not be able to grasp it in its entirety, and therefore to perceive its simplicity, and may mistake for it some specious counterfeit which appears to bear a simple aspect." P. 345.

The next character that we propose to introduce to our readers is that of Boerhaave's school-fellow, and almost equally distinguished cotemporary, Dr. MEAD.

The Rev. Matthew Mead, the father of Dr. Mead, was one of the 2000 ministers who were ejected from their livings on St. Bartholomew's day, by the Act of Uniformity, during the profligate reign of Charles II. Up to that time, he had been parish-minister of Stepney, and, after his ejectment, he continued to preach to a congregation of Non-conformists in the same place; and there his son Richard was born Aug. 11, 1673. Ten years after this date, the aged minister, being accused of disloyalty, was obliged to retreat into Holland; but, as he had a handsome fortune, this expatriation did not prevent him from giving his numerous family a liberal education. When sixteen years of age, Richard, one of 13 children, was sent to Utrecht, where he studied three years under the celebrated Græevius. Subsequently he went to the university of Leyden, where he attended the lectures of Pitcairn on the theory and practice of medicine, and those of Hermann on botany. Having completed his medical education, he made the tour of Europe, in company with Dr. Pellet, afterwards president of the London College of Physicians. At Padua, in 1695, he took the degree of Doctor; and, after visiting Rome and Naples, he returned to England in the course of the following year, married, and commenced practice at Stepney. In 1701, he published his work on Poisons. It has been remarked that there is in it a marked degree of reserve in speaking of certain deleterious substances, in consequence, it is believed, of the prevalence of secret poisoning in those days. He afterwards wrote on the Influence of the Sun and Moon upon the Human Body, and presented to the Royal Society an analysis of Bonomo's letter on the cutaneous worms which generate the Itch. In 1703, he was chosen physician of St. Thomas' Hospital, and was appointed by the Company of Surgeons to read anatomical lectures in their hall. In 1707, the university of Oxford conferred a doctor's degree upon him, and in 1716 he became a fellow of the College

---

* By some the name ΙΗΣΟΥΣ is derived from ιαομαι, ιησομαι, medeor, sano.

of Physicians. Mead was now in extensive practice, and had a warm and firm friend in Dr. Radcliffe, to whose practice, and house in Bloomsbury Square, he succeeded at his death. The day before the death of Queen Anne, Radcliffe being confined to the house with the gout, Mead was summoned to visit the royal patient. In 1721, he was deputed to superintend the inoculation of some condemned criminals ; the experiment succeeded, and the *operés* were set at liberty. In 1727, he was made physician to George II., whom he had served in that capacity whilst he was Prince of Wales ; and he had afterwards the pleasure of seeing his two sons-in-law, Drs. Nichols and Wilmot, his coadjutors in the same eminent station. In 1747, he published a treatise on Small-pox and Measles, in Latin. He also wrote a short discourse concerning Contagion, &c. ; in which he gave directions for a system of medical police, with the view of preventing the spread of the Plague which, being then at Marseilles, had caused great alarm in England.

Dr. Mead was a staunch whig, and had considerable influence with the then dominant part in the state. His generous use of this influence in the case of Dr. Friend was a splendid example of magnanimity and friendship.

"Dr. Friend had been committed to prison on suspicion of treasonable practices on behalf of the House of Stuart. Mead made many attempts to procure his discharge, but in vain, till being called in to attend Sir Robert Walpole, he made his friend's release the *sine qua non* of his attendance. The minister surrendered, and Friend was liberated; and, at an entertainment given at Mead's house to celebrate this event, the generous host put into the hands of his friend a bag containing 5000 guineas, being the amount of fees which he had received for him during his incarceration." P. 225.

Mead was a remarkably prosperous man. It has been said of him that, of all physicians who ever flourished, he gained the most,* spent the most, and enjoyed the highest favour during his lifetime, not only in his own, but in foreign countries. He was a munificent patron of literature and the arts, and was intimately acquainted with the leading men of talent of the day. With Boerhaave he long kept up a constant correspondence. Garth and Arbuthnot were his chosen friends. Pope was a frequent guest at his table, and has sung his praises in the well-known lines—

"Alive by miracle, or what is more—
Alive by Mead."

Young, too, has celebrated the medical skill of his friend and physician :

How late I shuddered on the brink ! how late
Life called for her last refuge in despair;
That time is mine, O Mead ! to thee I owe.

His charity and hospitality were large and ample, on a scale indeed of princely generosity. In his house in Great Ormond Street, he had a spacious

---

* The average annual receipts of his practice amounted, for several years, to between six and seven thousand pounds, at a time when the value of money was much greater than in the present day.

gallery filled with the treasures of art and literature. The catalogue of his library contained 6592 separate volumes, and his pictures sold, after his death, for £3400. He had, moreover, splendid collections of statues, prints, drawings, coins, and articles of vertu. He corresponded with all the principal men of letters in Europe, and in the decline of his life he received an invitation to visit the King of Naples, and inspect the newly-discovered city of Herculaneum—an invitation his advanced age compelled him to decline.

It was principally to him that the several counties of England and our colonies abroad applied for the choice of their medical men, and he was likewise consulted by foreign physicians from Russia, Prussia, Denmark, and other countries.

Among his other munificent acts, he caused the beautiful and splendid edition of Thuanus' history to be published in seven volumes folio; and, by his generous interposition, Mr. Sutton's invention of drawing foul air from ships and other close places was carried into execution, and all the ships in his Majesty's navy were provided with this useful machine. In short, nothing pleased Mead more than to call hidden talents into light, to give encouragement to meritorious projects, and to see them executed under his own eye. It was he also who induced the wealthy Citizen Guy to found the hospital that bears his name.

In the deline of life, Mead composed his "Medica Sacra," or commentary on the most remarkable diseases mentioned in the Bible. His "Monita et Præcepta Medica," were published still later. He died in February 1754, in the eighty-first year of his age.

Professor Marx's letter to Dr. Mead opens thus:

"It is a common saying that the King never dies, because he is immediately replaced; with equal justice may we say that a great man never dies, because he cannot be replaced.

"Having known and admired you for years, I turn to you, the object of my highest veneration, as to an old acquaintance. That we have never seen each other is nothing to the point. The blind never see their parents, the deaf never hear a brother's voice, and many see and commune with one another daily without ever really knowing each other.

"When a person like me writes to another so late in the day, it is to be presumed that he has made himself acquainted not merely with the whole man and his achievements, but also with his 'times,' that chief of re-agents, and blowpipe of events. The most accurate analysis brings out to our view the finished scholar, the distinguished practitioner, and the man of noble mind. It is to address the last-mentioned alone that I take up my pen.

"Of the many things which I have heard related of you, nothing has so much pleased me as your friendly conduct towards your school-fellow Friend. He would have long lain in the Tower, to which his too bold speeches in 1722 against the government as a member of parliament had consigned him, had you not obliged the Minister Walpole to set him at liberty. Not satisfied with this, you handed over to him £5000, received as fees for him in the interval of his imprisonment.

"The pious reflections which this genuine scriptural occurrence* suggest are these, that you were not induced by envy to wish a fellow-physician a thousand

---

* "In the 'Medica Sacra,' which you wrote after fifty years' practice, you say, miles off, or even in the land of shadows, but that you were desirous of having him near you, and that you gave up to him a sum for fees which in any other country would scarcely be accumulated during a long life, even with the help of government salaries, patrimony, and every legitimate service." P. 228.

After some very pertinent observations on the jealousies and unhandsome dealings that are but too frequent among medical men, from one to another, Professor Marx very pointedly remarks : "Discord and strife are unworthy of the science of healing ; war is only a disease.* Harmony and open honesty should be the symbol of professional intercourse. Hogarth, indeed, in his 'Analysis of Beauty,' speaks of the serpentine line as the line of beauty ; but in common life the straight line is to be preferred to the crooked one."

"It is not necessary that a man should sacrifice his nature, one can be independent without giving pain. Neither need we look upon active restless characters as dangerous, their excitability works for those who travel in beaten paths, like sour leaven or like caprification. For instance, a wild fig tree is set in the neighbourhood of the cultivated trees, that the insects from the wild one may pierce the buds of the others, and cause them to ripen more quickly." P. 231.

We have then, towards the close of the letter, some very flattering compliments addressed to England, and the character of its people. Gracefully commemorating us and our country as

> "This happy breed of men, this little world,
> This precious stone set in the silver sea."
> (Rich. II., Act ii.)

Marx alludes, with evidently cordial pleasure, to an incident which occurred to him in his visit (of which he has given a description in his former work, "Erinnerungen an England") to our shores.

"The victory of the will over matter is there everywhere apparent. Amongst my agreeable recollections, I place a stroll in the neighbourhood of London as far as Highgate with a friend, where at leisure we enjoyed the influence of retirement. You will remember that this was the place where, at a former period, the greatest thinker of England was struck by the angel of death on the first day of Easter, 1626. Lord Bacon was riding there with his household physician, Dr. Wilberborne. Snow was on the ground, and an idea struck him that it might be used to preserve meat from putrefaction like salt. Immediately they dismounted, went into a cottage close by, bought a fowl, had it drawn, and then stuffed it with snow. In this occupation Bacon became so unwell, that he was immediately afterwards compelled to go to bed, and in a few days after expired.

"The peculiarity and remarkable character of the natives occasioned me to make a reflection from which I will no more wander. There appears to be in England a kind of necessity to know the contrary side of everything which is

---

in the commencement of the preface : 'Scripta sacra, ut hominem Christianum decet, frequentius evolvi;' and at the end of the same : 'Fidem Christianam ab omnibus suis cultoribus id in primis exigere liquet, ut quaevis humanitatis ac benevolentiæ officia sibi in vicem praestent."

* "Benjamin Rush has written (Natural History of Medicine among the Indians—in his Inquiries, 5th Ed. vol. i., Philadelphia, 1818, p. 66) : 'War is nothing but a disease, it is founded on the imperfection of political bodies, just as fevers are founded on the weakness of the animal body.' "

revered and admired. They are as eager for the caricature as for the original. This seems to brace them like a cold sea-bath against a diseased sensibility. Their seriousness calls forth humour, their spirit of contradiction, satire. Out of the great they extract the ludicrous, not to trample it to the dust, but to survey it on the other side, to preserve themselves from over-valuing it, and to maintain a fitting moderation with regard to it.

"It is to you that the medical body is in a great measure indebted for the high esteem which they enjoy in that kingdom, and as there is an invisible church in science as well as in religion, even a foreigner may on this account be grateful to you." P. 233.

Dr. Mackness's observations on this letter to Dr. Mead are chiefly occupied with comments on the duties of medical men towards each other. He shews, by several examples, the evils of those petty rivalries and unworthy disputes that have, alas! in all times dishonoured, nay sometimes have even degraded, the noble profession to which we belong. Among other illustrations, he contrasts the high-minded feeling of generous friendship that existed between Dr. William Hunter and Cullen, with the unhappy controversy that existed between Dr. Leeds and Dr. Fothergill; the conduct of the latter on this occasion having, it must be confessed with regret, left a blot on his otherwise fair and dignified reputation. He very justly remarks that, "the only real cure for the hydra-headed evil of medical jealousy we believe to be in the elevation of view and purity of purpose of the individuals themselves. He who looks most on the Whole and to the Future is least likely to be taken up with the squabbles of the present."

For the particulars of the following biographical notice, we are indebted to the excellent little work, "The Life, &c.," whose title is affixed to this article.

GEORGE CHEYNE was born in Scotland in 1671. Little or nothing is known of his family; but he himself has gratefully acknowledged the advantage of having enjoyed "the instruction and example of pious parents." Being originally intended, like Boerhaave, for the ministry, he received a liberal education. While a tutor in a gentleman's family, he was induced to turn his thoughts to the study of Medicine by the celebrated Dr. Pitcairn, of whom mention has been already made, and whom he always loved to call "his great master and generous friend." His early predilection for the abstract sciences prepared him to adopt with energy and zeal the doctrines of the Mathematical or Mechanical School that Dr. Pitcairn had mainly contributed to introduce and render popular in this country. Having completed his medical studies (probably) at Edinburgh, he removed to London about the age of thirty, and soon after commenced practice as physician in the Metropolis. His account of himself at this time is very amusing. Hitherto he had been a sober, temperate, hard-working student; now he became the jolly frequenter of taverns and coffee-houses, with the view, it would seem, of acquiring notoriety, and enlarging the circle of his friends. "I found," says he, "the bottle-companions, the younger gentry, and free-livers, to be the most easy of access, and most quickly susceptible of friendship and acquaintance; nothing being necessary for that purpose, but to be able to eat lustily, and swallow down much liquor; and being naturally of a large size, a cheerful temper, and tolerable lively imagination, and having, in my country retirement, laid in store of ideas and facts; by these qualifications I soon became caressed by them, and grew daily in bulk, and in friendship with these gay gentlemen and their acquaintances.

"I was tempted to continue this course, no doubt, from a liking, as well as to force a trade, which method I had observed to succeed with some others; and thus constantly dining and supping in taverns, and in the houses of my acquaintances of taste and delicacy, my health was in a few years brought into great distress by so sudden and violent a change. I grew excessively fat, short-breathed, lethargic, and listless."

The result was a severe attack of intermittent Fever, followed by symptoms of threatened Apoplexy. When these at length subsided, he fell into a gloomy, hypochondriacal state, which, in the hands of an all-gracious Providence, was made the turning-point of his future welfare and happiness.

Previous to this period, Dr. Cheyne had brought his name before the public by two or three works which excited a good deal of attention at the time. In 1702, he published anonymously his "New Theory of Fevers," in vindication of the Iatro-mathematical views of his friend Dr. Pitcairn. To a modern reader, it exhibits a strange appearance; for, on turning over the leaves, it would seem to be a mathematical, rather than a medical work, being full of *postulates, lemmata, scholia, propositions, corollaries, geometrical figures, &c.* Such was the fashion of the age. It reached a fourth edition in 1724.

"His next sally," (1703) to use his own phrase, "was a book of abstract geometry and algebra, entitled 'Methodus Fluxionum Inversa,' brought forth in ambition and bred up in vanity." This involved him in a dispute with De Moivre, a celebrated French mathematician: and, as is the case with most learned disputes, the controversy on this occasion gave rise to a good deal of personal acrimony, at least on the part of our author.* He,

---

* The reader will be gratified, we should think, with the following observations, in which Dr. Cheyne expresses his estimate of the value of mathematical and other abstract studies, to the medical men more especially.

"To own a great but grievous truth, though they may quicken and sharpen the invention, strengthen and extend the imagination, improve and refine the reasoning faculty, and are of use both in the necessary and the luxurious refinement of mechanical arts; yet, having no tendency to rectify the will, sweeten the temper, or mend the heart, they often leave a stiffness, positiveness, and sufficiency on weak minds, much more pernicious to society and the interests of the great end of our being, than all the advantages they bring them can recompence. They are indeed edge-tools, not to be trusted in the hands of any, but those who have already acquired an humble heart, a lowly spirit, and a sober and teachable temper. For in others they are very apt to beget a secret and refined pride, an over-weening and over-bearing vanity, (the most opposite temper to the true Gospel spirit, which, without offence, I may suppose to be the best disposition of mind,) that tempts them to presume on a kind of omniscience in respect of their fellow-creatures, that have not risen to their elevation; and to set up for an infallibility, or at least a decisive judgment even in matters which do not admit of a *more* or *less*, (their proper object,) of which kind whatever relates to the Infinite Author of our being most certainly is." P. 16.

however, more than atoned for his error afterwards, by the honourable manner in which he apologised for his conduct.

"The defence of that book," says he, "against the learned and acute Mr. Abr. De Moivre, being written in a spirit of levity and resentment, I most sincerely retract, and wish undone, so far as it is personal or peevish, and ask him and the world pardon for it; as I do for the defence of Dr. Pitcairn's 'Dissertations' and the 'New Theory of Fevers,' against the late learned and ingenious Dr. Oliphant. I heartily condemn and detest all personal reflections, all malicious and unmannerly terms, and all false and unjust representations as unbecoming gentlemen, scholars, and Christians; and disprove and undo both performances, as far as in me lies, in everything that does not strictly and barely relate to the argument." P. 17.

In 1705, Dr. Cheyne brought out his "Philosophical Principles of Natural Religion;" a work that seems to have been a good deal esteemed; as it was used, he informs us, at both Universities as a text-book for the students.

It was probably at this period that our author was arrested in his course of convivial dissipation by the hand of sickness. The fever and threatened apoplectic seizure had left him a prey to severe headaches, giddiness, and lowness of spirits, with a broken and cachectic constitution into the bargain. As a matter of course, he found himself speedily deserted by his former boon-companions, to whom his fund of wit and lively conversation had rendered him peculiarly acceptable. His health, indeed, had suffered so much that he was obliged to retire into the country.

"While I was thus forsaken by my holiday friends, and my body was, as it were, melting away like a snow-ball in summer, being dejected, melancholy, and much confined at home, by my course of mineral medicines, and country retirement, I had a long season of undisturbed meditation and reflection, (my faculties being then as clear and quick as ever,) which I was the more readily let into, that I concluded myself infallibly entering into an unknown state of things." P. 22.

The result was indeed a happy one to him, as to many others who have been suddenly chastened by sickness and pain, at a time when all was gay and prosperous about them. What seemed an affliction is made a blessing. How full of deep and solemn truth are these words of our great bard!

"There is a soul of good in all things evil,
Could men observingly distil it out."

Quiet reflection brought Dr. Cheyne to a sense of higher duties and nobler enjoyments than he had yet thought of. Then it was that he became more calm and cheerful, and his gloomy thoughts forsook him. These are his own words: "I never found any sensible tranquillity or amendment, till I came to this firm and settled resolution in the main: viz. *To neglect nothing to secure my eternal peace, more than if I had been certified I should die within the day; nor to mind any thing that my secular obligations and duties demanded of me, less than if I had been insured to live fifty years more.*" A sentiment which Samuel Johnson took pleasure in repeating: it deserved, said he, "to be imprinted on every mind."*

* There is another of Dr. Cheyne's sayings that has been rendered familiar to

Dr. Cheyne's health, although much improved by his retirement into the country and the low regimen he pursued, was not yet completely restored. He was therefore advised to try the Bath waters. He did, and found considerable relief for some time. About this time, he commenced living upon a diet of milk and vegetables. From this system he derived great benefit, diminishing much in corpulency (for he was of enormous size), and recovering his strength and activity entirely. At length he was enabled to resume his professional avocations; dividing his time between Bath and London, and devoting his attention chiefly to the treatment of chronic, and especially to low and nervous, cases. In course of time his practice must have become very prosperous, as he was one of the leading physicians at Bath under the fashionable reign of Beau Nash. How long he kept upon the milk diet, is not known; but it would seem that about the year 1712, he began to resume the use of animal food; and, for several years afterwards, he appears to have continued in good health.

In 1715, he published his second part of his "Philosophical Principles of Religion" In it, he applied mathematical reasoning to the discussion of theological doctrines, as he had previously done to the examination of medical questions; and with nearly equal success.*

It was in 1720, that he commenced that series of popular medical works which brought him so much reputation in his life, and by which he is now chiefly known. The first of these was the "Essay on the Gout and Bath Waters." It proved very successful, passing through seven editions in six years. Its concluding observations are as follow:

"As it is only the rich, the lazy, the voluptuous, who suffer most by the Gout, (I mean *acquired* Gouts, and those hereditary ones enraged by luxury,) so those only who have spent their life-time under its tortures best can tell, what astonishing miseries wealth and vice bring upon human kind. When the gouty humour has seized upon all the noble principles of life, when it has broken, subdued, and obstructed all the fine pipes and slender passages, in whose openness and soundness all the exquisite sensations, all the delicate usages of the animal faculties consist; when nothing but pain, and melancholy, frightful ideas, horrible dreams, and black despair, remain; who would not have parted with the richest delicacies, the most delicious wines, and the most enticing vices, for a plain simple diet, an useful laborious life, freedom from pain, and a good conscience? Temperance only, divine, innocent, indolent, and joyous temperance, can cure or effectually relieve the Gout: for let us, or our brethren the *quacks*, brag what we will,

'Tollere nodosam nescit medicina Podagram.'" P. 36.

Notwithstanding his own precepts, it would seem that our author must have gradually lapsed, in some degree at least, into his early habits of free living; for we find that in 1723, after having become enormously lusty

---

us by being quoted with approbation by Johnson: "Everything is best as it has been, except the errors and failings of our free wills."

* Among other works of that day was one entitled "Theologiæ Christianæ Principia Mathematica," from the pen of the Rev. John Craig, Vicar of Gillingham, Dosetshire. One of the propositions stands thus:—Valor verus expectationis ad obtinendam voluptatem a Christo promissam est infinito major vero valore expectationis obtinendi voluptatem vitæ presentis.

(weighing more than 32 stone !) he had a severe attack of fever and erysipelas. His health continued infirm for a year and a half. He again had recourse with excellent effects to his milk and vegetable diet, and to this regimen he almost entirely confined himself during the remainder of his life.

What adds much to the interest of Dr. Cheyne's biography is the exhibition it, more than once, affords of strong-hearted diligence and activity of mind struggling with and overcoming the depressing effects of bodily infirmity. While scarcely expecting ever to recover from his late illness, we find that he was busily engaged in writing his well-known "Essay on Health and Long Life," one of the most successful medical works that was ever published. It ran through seven editions in the course of two years. It was translated into Latin, French and Italian. It has been reprinted in this country within the last 20 years. The charm of the work consists in its being the faithful and well-told record of the author's personal experience; and, being written in a fine bold manly style alike of thought and language, and replete with sound maxims of moral as well as professional wisdom, it still deserves, and indeed retains, a place in the physician's library.

"If this work added much to Dr. Cheyne's popularity and reputation, it also exposed him to a storm of ridicule and banter, chiefly on account of the abstemious regimen recommended, which no doubt in the eyes of the world contrasted strangely enough with the peculiarities of his personal appearance. 'Some good-natured and ingenious retainers to the Profession,' (says he,) 'on the publication of my book of 'Long Life and Health,' proclaimed every where that I was turned mere enthusiast, and resolved all things into allegory and analogy, advised people to turn monks, to run into deserts, and to live on roots, herbs, and wild fruits; in fine, that I was at bottom a mere leveller, and for destroying order, ranks, and property, every one's but my own: but that sneer had its day, and vanished into smoke. Others swore that I had eaten my book, recanted my *doctrine and system,* (as they were pleased to term it,) and was returned again to the devil, the world, and the flesh. This joke I have also stood. I have been slain again and again, both in verse and prose; but I thank God I am still alive and well." P. 59.

In 1733, Dr. C. published another of his most celebrated works, entitled "The English Malady, or a Treatise of Nervous Diseases of all kinds, as Spleen, Vapours, Lowness of Spirits, Hypochondriacal and Hysterical Distempers, &c." It is in this that he has given an account of his own case (from the narrative of which most of his personal history is derived), and also the very remarkable one of Col. Townshend, who seemed to have the power of suspending the action of the heart by a mere effort of his will.

In 1739 appeared his "Essay on Regimen, together with five Discourses, Medical, Moral and Philosophical;" but it failed to interest the public: "the mixture of mathematical language tends to render the work repulsive, and in some places unintelligible to all but mathematicians." Three years subsequently he published his last work, entitled "The Natural Method of curing the Diseases of the Body and the Diseases of the Mind depending on the Body." This was much more successful than the preceding one. It was dedicated to Lord Chesterfield, whose letter of acknowledgment we should have liked to have transcribed entire, had our limits allowed. Here is its closing paragraph:—

"I read with great pleasure your book, which your bookseller sent me according to your directions. The physical part is extremely good, and the metaphysical part may be so too, for what I know: and I believe it is; for, as I look upon all metaphysics to be guess-work of imagination, I know no imagination likelier to hit upon the right than yours; and I will take your guess against any other metaphysician's whatsoever. That part which is founded upon knowledge and experience, I look upon as a work of public utility, and for which the present age and their posterity may be obliged to you, if they will be pleased to follow it." P. 102.

This last work is more exclusively practical than perhaps any of Dr. Cheyne's other writings. It contains many valuable observations on the treatment of diseases, and on the management of our moral faculties, that will well repay an attentive perusal. Some good extracts are given in the "Life." The author did not survive its publication more than a twelvemonth. He died on the 13th of April, 1743. In one of the papers of that day, he is called "a learned physician, a sound Christian, a deep scholar, and a warm friend." The following is the fair and judicious estimate which the writer of the "Life" has taken of Dr. Cheyne's medical writings.

"The class of '*popular*' medical books is almost universally condemned by the more respectable members of the Profession, and for the most part deservedly; as in many cases these works are likely to lead to mischief by giving that '*little* learning,' which in Medicine is peculiarly 'a dangerous thing.' The chief objection, however, against them arises not so much from the fact that they are addressed *directly* to the public, instead of through the medium of the Medical Profession, (for the highest truths may be conveyed in a popular form,) as from the general character of the books themselves, which for the most part bear evident marks of the incapacity of the writers. Occasionally, however, there have arisen men, like Cheyne and Tissot,* who, while they have proved that they are fitted to instruct their professional brethren by their purely scientific writings, have nevertheless not disdained to endeavour to supply the public with really *good* books of popular medicine, instead of the worthless or dangerous trash they so greedily devour. Dr. Cheyne's writings, which were much read and had an extensive influence in their day, procured him a considerable degree of reputation, not only with the public, but also with the members of his own Profession. If they present to the reader no great discoveries, they possess the merit of putting more prominently forward some useful but neglected truths; and, though now probably but little read, they contain much matter that is well worth studying, and have obtained for their author a respectable place in the history of Medical literature." P. 129.

---

* To these two names, how well may we now add that of one not less eminent in his day than either—the author of the admirable "Change of Air," the "Essay on Indigestion," the "Stream of Life," &c. not to mention the more classic and standard work on "Tropical Climates." It would be easy to point out several features of resemblance in the professional careers of Dr. Cheyne, and of our late dear and venerated friend. One will suffice. We have seen that the former composed his first successfully popular work, while labouring under bodily and mental depression. So it was with Dr. James Johnson. His "Essay on Indigestion," that first fairly established his practice as a leading physician, was suggested, we might rather say, prompted by personal experience of the wretched hypochondriasis which the malady is so apt to induce. *Vide* the Biographical Sketch in the Medico-Chirurgical Review for January, 1846.—*Ed.*

The preceding biographical sketch having grown under our hands into somewhat larger dimensions than was intended at first, we must curtail our notices of Professor Marx's letter to Dr. Cheyne, and of Dr. Mackness' comments upon it. From the former we select the following passage :—

" There are two things which I attribute to you, and which conspire to awaken confidence in your character and judgment: the one is your decided aversion to all personal strife, the other your well-grounded recommendation of water-drinking. The accidental remark that abstinence tends to produce serenity of mind, is one that I can heartily subscribe to.

" Should I ever take upon me to publish my sentiments on dietetics, my rules for the promotion of happiness will be based on a few propositions; tending to manifest that health is a virtue, cheerfulness a duty. I would show that one cannot be too deeply impressed with the feeling of aversion to sickness, for a sickness which is incurable is not only a clog, but a lie against our destined lot. The *valere aude* I would put forth as the general salutation." P. 36.

" My efforts will be limited to the endeavour to exhibit the simplest means by which to preserve continual cheerfulness, to have the body not for an accessory in evil, but a friend, and, if necessary, to lay out one's life to some useful purpose.

" It must be plainly confessed that most are unhappy on this account, namely, that they live in dread of the possibility of misfortune, give up too readily to it when it occurs, and do not sufficiently notice its palliative circumstances.

" The full experience of happiness appears, to the anxious and timid mind, something so doubtful that they pray that it may not be sent. They consider not that prosperity, that is, a wise self-confidence, awaits the bold.

" Regrets accompany most events; we smile at the devotee who lacerates himself amongst the tombs, and yet we ourselves weep over the experience which has been collected in the grave of the past. As mendicants live by their sores, so we suck consolation out of our inward weakness.

" But help may be sought in a very different direction, by being strict in self-government, the master of our own house, and own person: *nunquam retrorsum,* that is to say, never gloomy. Cheerfulness is to be sought in free intercourse with Nature, with men, or with books; and the purest enjoyment is preserved, like seed-corn in the earth, to spring up in gloomy weather.

" The saying that a man's stomach is his destiny is essentially true; the best race-horses take the least food. *Mors in olla* means, not death iu the pot itself, not in its leaden or copper lining, but in its contents; he who fills his stomach too full, must not wonder if sometimes his heart is full also.

" Physicians must perseveringly enforce a reasonable system of dietetics; by this means they will not merely contribute to invigorate life, but to prolong it." P. 38.

On the question of Dietetics, as suggested by Professor Marx, Dr. Mackness judiciously observes :—

" It is, we believe, a subject of immense importance in medical practice, and one which is far from receiving commensurate attention at the present time. Strict attention to dietetics has been one grand means by which empiricism has attained success—a success which has been favoured by a corresponding neglect on the part of established practitioners. Hydropathy and Homœopathy owe much of their celebrity to this source, and whenever men of talent and judgment have condescended to avail themselves of this simple means, the results have been highly beneficial. Our subject leads us rather to inquire what is the moral bearing of the question. And here we think it distinctly the duty of the medical man to use all his influence with his patients to adopt and persevere in such systems of di[illegible] as he may have reason to think will, in their particular cases, most tend to preserve the *mens sana in corpore sano*, not allowing them to suppose it is sufficient to do so during the period of sickness or convalescence, but habitually to look upon such care as a preventive of disease, and enforcing that view which Professor Marx takes, that it is a duty incumbent upon every one to take a rational care of his health, as the great means by which he may be enabled to perform efficiently those duties to which Providence has appointed him. Persons will often pay much attention to these views, when urged with all the weight of medical authority, who would laugh to scorn the very same opinions if proceeding from some judicious non-medical friend. Most persons desire health, and would make sacrifices to procure it; but many would submit more easily to severe remedies to remove disease than to a little habitual self-denial to preserve health, and the very simplicity of the means operates against their recognition. They cannot believe that a little indulgence in one thing one day, and in another at some other time, can do them any harm: and thus the medical man is often obliged to have recourse to strict and definite rules, rather than to lay down general principles." P. 40.

We come now to the fourth and last character that we have selected; one, which the graces of benevolence, friendship, and charity will render very attractive.

JEAN NOEL HALLÉ was born at Paris on the 6th of January, 1755. In early life, he had the great advantage of the advice and instruction of his maternal uncle, Aimé Charles Lorry, one of the most distinguished and intelligent physicians in France at the close of the last century. He took his first degree in 1776, after displaying so much ability in his examinations, that the founders of the Royal Society of Medicine requested him to be a companion of their labours, even before he had received his doctor's diploma.

"This precocious honour," remarks M. Cuvier in the *eloge* pronounced by him on the character of Hallé in the Institute of France, "afterwards prevented him from obtaining the title of Doctor Regent in the Faculty of Medicine. Fourcroy and several other men of first-rate talent suffered the same disgrace from the same cause. This puerile jealousy which had led the Faculty to regard the Royal Society of Medicine as a rival body, had also induced it to vow an implacable hatred to those of its own members who had consented to belong to it. When it is remembered what antipathies this jealousy excited amongst the physicians of the capital, and the ridiculous dissensions and odious satires it produced, it may give a favorable idea of the mildness of character and modesty of Monsieur Hallé, and also the regard which those qualities inspired, that while the highest reputations were not respected in the writings of the day, he was less vituperated than any of his associates. Elevated indeed beyond all intrigue, and only desirous of increasing his professional knowledge by all those aids of science which could assist hlm, and neither pluming himself upon the success of his discoveries, nor seeking popular applause, he neither wounded the vanity, nor alarmed the interest of any one. The study of medicine appeared to him quite sufficient to occupy the whole of life. In his view of the subject, there was nothing which could influence man morally or physically that did not belong to this noble science, and he therefore manifested so disinterested a feeling towards it, as to regard every means beneath his notice for obtaining public confidence, except such as were truly desirous of it. He was therefore continually to be found at the bed-side of the sick, watching the progress of disease, or in his own room, engaged in the study of practical medicine, chemistry, and even of political economy, as far as it related to the benefit of the different classes of society. Nor did he neglect anatomy and physiology, but still regarding these sciences as subservient only in their relations to the health of men generally or of individuals." P. 49.

He contributed several papers on Practical and Hygienic Medicine to the Memoirs of the Royal Society of Medicine. In 1784, he published an edition of Lorry's work, entitled, "*De præcipuis morborum mutationibus et conversationibus;*" and subsequently he edited the writings of Bordeu upon the Glands and cellular tissue.

His disinterested humanity to the sick poor of Paris was so universally known that, when the National Convention ordered the nobles (and Hallé's father and grandfather, having each received the badge of St. Michael, were consequently considered as such) to quit the metropolis, he was of course included in the sentence, and was exempted from the penalty, only upon the ground of his being the kind-hearted physician of the destitute and afflicted. It was at this time too that he displayed so much heroism and generosity of friendship in aiding the escape of many who, from whatever cause, had fallen under the suspicion or displeasure of the existing government. "He penetrated into the prison of Malesherbes, giving him consolation, and receiving his last adieus. At the Lyceum of Arts he drew up the petition for the pardon of Lavoisier, and during the two years which may be called the age of misery and shame, he occupied himself assiduously in performing a thousand other services to the unfortunate, for doing which the principal condition he required was secrecy."

When Fourcroy in 1794-5 was called upon to re-organise the school of medicine in Paris, the chair of Physique Medicale and Hygiene was conferred upon Hallé; and in the following year, on the establishment of the Institute of France, he was named a member of the department of medicine and surgery.

In 1806, Corvisart, who was entirely occupied with his duties as physician to Napoleon, chose him for his colleague at the College of France, and soon after gave up the post altogether to his care. This distinguished physician, when he bequeathed the portrait of Stoll to Hallé, said in his will that he left it to that physician whom he most esteemed.

Among his labours at the Society of Medicine, in whose welfare he continued to take an active interest, we may particularly mention his steady and zealous advocacy of Vaccination. He contributed much to its general introduction into France. Italy, too, owes to him a particular remembrance in this respect. He was employed in 1816 to introduce it into the states of Lucca and Tuscany, and his success in this mission tended to render his name very popular in those countries.

Hallé was a man of very great erudition, an admirable scholar, and deeply versed in most departments of physical science. In the practice of his profession, he was generous and liberal even to a fault. We have already had occasion to allude to his kindness and attention to the poor and suffering. Not content with being ever ready to aid them by his medical skill, he took pleasure in relieving from his own purse their wants and necessities. Many a person, who could not afford to pay, found, after his convalescence, that all the expenses occasioned by his illness had been defrayed, and only then by careful enquiry discovered that his physician had provided for all.

"Returning home," says his eloquent panegyrist, "at the close of the day, exhausted with fatigue, it was, perhaps, announced to him that a lady wished to consult him; he would desire his servant to advise her to go to one of his medical brethren, a message is returned that she cannot do so, for she has nothing to pay him with; this is an appeal he cannot withstand. 'Oh, in this case, then,' replies he, "I have no choice, I must attend to her.' His generosity was ever prominent; he invariably gave the entire profits of his works to the young persons who rendered him assistance in collecting materials for them. Having been charged with the task of editing a new pharmacopœia, he employed the sum which was allowed by government for this purpose in the completion of the hall of the Faculty of Medicine. Happy in the good that he did, happy in his success, happy in his family, M. Hallé seemed to possess what is beyond every other earthly blessing; his health was robust, and although occasionally troubled with a little fulness of blood, prompt bleeding immediately relieved him, but all at once he found himself suffering from stone in the bladder. Yet even in such painful circumstances, when most persons would have been only occupied about themselves, his unbounded charity was still active; before he underwent the operation of lithotomy, though suffering great pain, he yet went to see some poor persons whom he supported, fearing that his long absence might appear to them an act of forgetfulness." P. 56.

The operation was successfully performed; but a congestion of the lungs followed, which proved suddenly fatal on the 11th of February 1822. Hallé was 68 years of age at the period of his death.

The letter addressed to him by Professor Marx is one of the most beautiful in the series; alike felicitous in conception and graceful in expression. We cannot resist the pleasure of giving it entire.

"The history of your life, can well attest the sufferings which a medical man of fine feelings has to endure; and, therefore, I shall find indulgence, if I venture to impart to you some sentiments respecting the trying hours of our profession. No one will better understand me.

"It was but the painful sequel to a much tried life, when at the age of 68, shortly before your death, you submitted to the anguish of lithotomy, which nothing but the force of resignation could have enabled you to sustain with your habitual gentleness.

"To what extent you were the protector, the friend, and the helper of the poor, was shown in a time when a part of mankind had ceased to be human.

"It was yours to experience what it is to serve others with our whole soul, and yet to be misjudged by them, and then, as it were by the majesty of innocence, to compel esteem from the madman.

"The hound set on against his benefactor often recognizes him in the moment of attack, and instead of tearing, covers him with caresses.

"In the tumult of unbridled passion, you were enabled to preserve calmness of mind for yourself and others.

"To you it was permitted to visit Malesherbes in his imprisonment, and to receive his farewell; you drew up the petition for Lavoisier.

"Could the stones of Paris speak, they would testify that you alone wiped away the tears of the sorrowing.

"Every project in medicine tending to the benefit of society might safely reckon on you as its patron and protector. How untiringly did you contribute to the spread of Vaccination!

"You have kept no record of personal sacrifices and thanks received; where the deficit lay it is easy to divine.

"You acted benevolently with a full participating heart; you were rather surprised when in any case gratitude followed, than shocked when it was wanting.

"Franklin relates that he lent a sum of money, and when the debtor would have returned it to him, he requested him to lend it to some other person in similar need, and so on continually. Thus did you consider property as a deposit —a debt to be discharged.

"But if the physician works with his mind as Fenelon teaches than men ought generally to work, is it not true that the burden of the profession, or rather the addition of selfishness, often presses like lead on his heart?

"Those, who are conversant only with business or mechanical employments, can scarcely imagine what a heavy heart the medical man takes with him out of the house of death.

"There are indeed physicians who look upon disease and death merely in the abstract, and who would seem to have to do not with the *sick* but with *sickness*, not with the *dying* but with *death*, who practise *lege artis*, and content themselves with common-place morality; with such I shall not trouble you.

"Neither does death awake any overwhelming compassion in cases where the cessation of suffering appears as a benefit.

"In such instances, sickness deals with the invalid as a gardener does with a tree which he wishes to transplant, and whose roots he therefore carefully loosens from the soil. The separation from their accustomed habits and relations takes place then so gradually that it comes to be considered like the natural result of preceding changes.

"But how is it when a dangerous illness falls like a rocket into the house, and now none but the physician can save? when the life sinks, not gradually and gently like the fluttering of a leaf before it falls from the tree, or the stopping of a watch, but when Nature, like a tragedian, seems to have compressed the most affecting scenes into the last act?

"Exhausted, returns the medical man to his house, solacing himself with the hope of forgetting the toils of the day, and renewing his strength in refreshing sleep; when lo! at midnight he is summoned to a child who is dying of croup. The parents welcome him as an angel from heaven; it is the first time for days that they have attended to any one but their own child; they hang breathless on his expressions; they scan his features to extract from them his thoughts; they draw hope from every question, every direction, every gesture; the mother smiles at him in half-desponding thanks because the child is quiet; the father in emotion grasps him by the hand; but the quiet is of short duration, the child can cough no more, it bends its head backwards, it stretches out its limbs convulsively to breathe—in vain, it expires.

"Who else is now the companion to the physician besides the groaning lamentations of the stricken parents?

"Should he hereafter lose a friend, one perhaps on whom he has cheerfully expended years of toil, self-denial, self-sacrifice, where can he turn for pity?

"From the furnace of his anxieties he is followed by the sighs only of those who intimately share them; few concern themselves about him, who is nevertheless harassed and worn down by his efforts to assuage the woes of others.

"Another practitioner goes with a heart oppressed with grief to his chamber; he is immediately called to a woman in labour, and compelled to perform the operation of craniotomy.

"Nor is it enough that in critical and decisive moments he draws, like the orphan boy in a lottery, he knows not whether a prize or a blank; that, like a swimmer, he has to struggle with the apparent dead; that, like a father confessor, he has to speak consolation at the very gallows,—no, he must pass the ordeal of ignorant and perverted judgments. In thus running the gauntlet of reproaches on the one hand, and envious joy on the other, he must sustain himself by his conscious innocence, as men who are undergoing operations or suffering pain bite a bullet to prevent them crying out.

"For the dying there is an Euthanasia, for the mourner a visit of condolence, but who concerns himself about the suffering physician? And yet he has most frequently to experience that in bereavements the tears of survivors become like aquafortis to his soul, and that powerlessness to save others curdles as it were his own blood.

"But I hear you exclaim, 'O desine renovare dolores!' and therefore I will cease to complain, and solace myself with the hope, that as in other respects there have been improvements in the condition of the medical man, so there will be also in regard to consideration shown to their feelings.

"He, who has read the letters of Zimmermann which appeared after his death, will remember that medical attendants in noble houses were formerly accommodated with a *seat* but not with a *chair*, and that domestic physicians were permitted to use riding horses but not leather bridles.

"There is no word of more frequent recurrence in Japan than 'Patience!' Golownin's journey and the narrative of his captivity in that country suffices to teach the European physician contentment. You indeed practised patience and resignation so thoroughly, that you may justly claim the palm of victory." P. 61.

The remarks of Dr. Mackness upon this admirable letter are worthy indeed of his theme:—

"The trials (of medical men) here described, may be chiefly classed under two heads—the *sorrows of sympathy* and the *sorrows of isolation.*

"The first of these, the *sorrows of sympathy*, especially pertain to the medical profession, and woe to him who enters into it without a full appreciation of his requirements in this respect, for his duty and conduct will then be full of inconsistencies. A medical man, more than any other individual, is called to drop in the words of consolation in the hour of trouble, for to him only is often confided the heart-sorrows of his fellow-creatures. What is a medical man worth who has no feeling for his patients, or who looks upon them merely as puppets, by working with which he is able to make up a certain amount of income! who has not a deep sense of the solemn responsibility of being, to some extent, the guardian of the lives and health, those dearest earthly blessings which constitute the happiness of his fellow-creatures, who is incapable of tasting the sweet pleasure of bringing ease to the sufferer—hope to the drooping—health to those who, without his aid, would sink under disease!" P. 62.

After showing that nothing save "high moral and religious principles of action" can sustain and direct the physician in the many and varied trials which his mission necessarily imposes upon him, and quoting a very beautiful passage from the correspondence of Dr. Lettsom, Dr. Mackness vindicates, with much good feeling, the character of the medical profession in point of disinterested humanity to the suffering poor.

"Who is first appealed to by the destitute in the hour of nature's suffering? Nay, who is most ready to attend on such occasions? It is the medical man. Wherever human misery exists, wherever pain is endured, in the lowest hovels of the poor, where disease and death, contagion with all that is offensive to the outward senses are present, there will be found the medical man, alleviating pain, soothing the sorrowful, smoothing the pillow of death, and offering consolation to the survivors; and often all this without the slightest expectation of reward. No class of the community, not even the ministers of religion, are called to make such immense personal sacrifices as medical men; nor is any class so little appreciated for their humanity and generous disinterestedness, at least by the public generally." P. 65.

Often indeed are his services, even when most harassing, not known; and, when known, not appreciated or even admitted. No one, therefore, has so much need as a medical man of looking for his recompence in the secret approval of his own conscience, and of Him who is greater than his conscience, and knoweth all things.

So much for the *sorrows of sympathy*; let us now see what Dr. M. has to say touching the *sorrows of isolation.* The following passage will shew his sentiments upon this subject.

"The other trials of feeling mentioned, peculiar to a medical man, seem to arise out of his isolated position. Few but those of his own profession can enter into his feelings, or sympathise with them, and when sickness comes near himself or his family, he has, less than others, the comfort of hope. He is so conversant with every symptom, every ailment, that his mind is apt to get into a morbid state through his very familiarity with disease, and hence it has often been remarked that medical men are bad patients. If it be, indeed, the fact that medical men receive less of general sympathy in the domestic and personal trials which befall them, it may, perhaps, arise from this, that they are supposed to be so sufficient for themselves that sympathy is deemed almost impertinent. Another and a sadder cause, when sickness befalls them in their own persons, is, that they are compelled to conceal it as much and as long as possible, lest they should be deemed unfit for their duties. There is, indeed, a sad want of gratitude and generous feeling sometimes displayed in this respect. On the first indication that a medical man's health is failing, persons will begin to relax in their adherence to him, and to look about for some new candidate, when a little patience and forbearance on their part might enable him, who has, perhaps, many a time stood beside their couch of suffering, to regain his exhausted strength, and preserve his position. A beautiful instance of consideration and kindness is mentioned in the life of the late excellent, and noble-minded Dr. Arnold, when he was attacked by the fatal seizure which deprived, in so brief a space, our country and church of one of their brightest ornaments; he objected to his wife's wish immediately to summon medical aid, that the hour was early, and that he did not like to disturb Mr. Bucknell who had been recently ill. We are far, however, from believing that such instances are singular." P. 69.

With respect to the last sort of trial to which medical men, more than any other persons, are exposed, viz. hasty and unfair censure, we have already seen how such men as Boerhaave and Cheyne felt and acted when calumniated or unjustly accused. Whoever has the "mens sibi conscia recti" within, need not fear the shafts of envy or malice. It will never do for a medical man, if he seeks to enjoy peace and comfort at home, to think much of all the ill-natured remarks, evil speakings or insinuations to which he may be exposed abroad, whether from patients or from his professional brethren. There is a way by which he can "make even his enemies to be at peace with him." We quite agree with Dr. Mackness, that the practice of the late amiable Dr. Hope—that of taking notes of every little circumstance which he thought capable of misconstruction, and obtaining the signatures of witnesses to vouch for their accuracy—is one not to be commended or imitated, at least as a general rule. It seems to indicate too little trust in the force of truth, and in the majesty of innocence. A much wiser and better advice is that of Dr. Mackness: "be patient under the trial, knowing that merit always outlives calumny, and strive, by cultivating the highest motives, to live above unjust censure."

And now we must quit the pleasant company of Professor Marx and his worthy translator; trusting, however, that, ere long, we shall have again to welcome their labours in the same agreeable path of authorship.

Had our limits permitted, it was our intention to have borrowed more largely from the pages of the "Life of Dr. Cheyne." Altogether, it is one of the most interesting—aye, too, and instructive—specimens of medical biography which we have met with for a long time. It forms one of a series in the course of issue under the editorial superintendance, we understand, of Dr. Greenhill of Oxford, so favourably known to the profession by his admirable edition of Sydenham's works, and by the discovery and publication of the "Anecdota Sydenhamiana." Already the lives of Sir James Stonehouse, Dr. Burder,* and Cheyne have appeared; and we are promised those of Boerhaave, Haller, Hey, Abercrombie and others, who have adorned our ranks by the moral graces, as well as by the intellectual excellencies, of their character. 'Tis a pleasing sign to observe a taste for professional biography springing up in our literature; few things are better calculated to keep alive a spirit of sound and healthy feeling among us than the occasional retrospect of the lives of the great and good. It is therefore with true pleasure that we find that the editor of our very talented cotemporary, the Dublin Medical Review, has commenced a series of sketches of the more distinguished of his countrymen. The Memoir of Dr. Mosse—the founder of that noble institution, the Dublin Lying-in Hospital—in the number for last November, is full of interest. What an example of intrepid unwearying philanthropy does it unfold!

* There is much, in the memoirs of this very estimable man, to awaken a medical practitioner to a proper sense of the duties of his responsible calling. We would specially direct the attention of the reader to the "Letters from a Senior to a Junior Physician" (which have already appeared in the Life of Dr. Hope), and also to the admirable letter of the late Dr. Abercrombie—whose praise is on everyone's lips—to Mrs. Burder on the death of her husband.

